# Clinical Efficacy, Tolerability and Safety of a New Multiple-Action Eyedrop in Subjects with Moderate to Severe Dry Eye

**DOI:** 10.3390/jcm11236975

**Published:** 2022-11-26

**Authors:** Anna Maria Roszkowska, Leandro Inferrera, Rosaria Spinella, Elisa Imelde Postorino, Romana Gargano, Giovanni Wiliam Oliverio, Pasquale Aragona

**Affiliations:** 1 Ophthalmology Clinic, Department of Biomedical Sciences, University Hospital of Messina, 98122 Messina, Italy; 2 Ophthalmology Department, Faculty of Medicine and Health Sciences, Andrzej Frycz Modrzewski Krakow University, 30-705 Krakow, Poland; 3 Eye Clinic, Department of Medical, Surgical Sciences and Health, University of Trieste, 34127 Trieste, Italy; 4 Department of Economics, University of Messina, 98122 Messina, Italy

**Keywords:** dry eye disease, cross-linked hyaluronic acid, trehalose, liposomes, sterylamine, osmolarity

## Abstract

*Background:* To assess the clinical efficacy, tolerability and safety of a new-generation ophthalmic solution containing cross-linked hyaluronic acid 0.15% trehalose 3%, liposomes 1% and sterylamine 0.25% (Trimix^®^ Off Health Italia, Firenze, Italy) (CXHAL) versus trehalose 3% (Thealoz^®^, Thea Pharmaceuticals, Clermont-Ferrand, France) (TRS) in subjects with moderate to severe dry eye disease (DED). *Patients and methods:* In this prospective, observational cohort study, 41 subjects with moderate to severe dry eye were enrolled and divided into two age- and sex-matched groups. Group 1 was treated with CXHA eye drops, and group 2 was treated with TRS eye drops four times daily for 2 months. All subjects were evaluated at baseline (V0) and at day 60 ± 3 (V1). The examination comprised Best Corrected Visual Acuity (BCVA) and Symptom Assessment in Dry Eye (SANDE). Tear osmolarity was evaluated using the TearLab Osmolarity System^®^; Keratograph 5M (Oculus, Wetzlar, Germany) was performed to assess tear meniscus height (TMH), fluorescein tear break-up time (TBUT) and corneal and conjunctival fluorescein staining and meibography; furthermore, slit lamp evaluation was performed for eyelid erythema and edema, conjunctival chemosis and hyperemia and Meibomian gland secretion quality. *Results:* All patients completed the treatment. BCVA remained stable in both groups, and no adverse events were reported. After 2 months, both groups showed statistically significant improvements for SANDE (*p* = 0.001 and *p* = 0.012, respectively), TBUT values (*p* < 0.001 and *p* < 0.001, respectively) and staining (*p* = 0.004 and *p* = 0.001, respectively) as compared to baseline values. Group 1 showed a statistically significant improvement in SANDE frequency and tear osmolarity (*p* = 0.02 and *p* = 0.001, respectively), whereas chemosis was significantly reduced in group 2. The amount of TBUT improvement was statistically higher in group 1 compared to that in group 2 (*p* = 0.041). *Conclusion:* A new-generation multiple-action ophthalmic solution was safe and clinically effective in the treatment of moderate and severe dry eye, with significant improvements in the main ocular surface parameters.

## 1. Introduction

Dry eye disease (DED) represents one of the most common ocular disorders, with the reported prevalence rate ranging from 5 to 50% and a peak after the fifth decade of life [1,2,3,4,5,6]. It was identified as a disorder of the Lacrimal Functional Unit (LFU), an integrated system comprising lacrimal glands, ocular surface, lids and sensory and motor nerves. Resulting in significant morbidity, DED remarkably impacts the visual function and patients’ quality of life [3,7].

DED may present clinically with a slight to severe ocular discomfort associated with different intensity signs and symptoms and visual acuity impairment. The two main pathogenic mechanisms, namely, aqueous deficient or evaporative, generally manifest contextually in the same patient such that it may be very difficult to understand the main mechanism responsible for it [1,7,8].

The definition of DED has changed over time, and the most recent one, formulated at the Tear Film and Ocular Surface Society International Dry Eye Workshop II (TFOS DEWS II), highlights the role of neurosensory abnormalities in the etiology of the disease, where the multifactorial aspects of the DED are driven by the loss of the homeostasis of the tear film [8].

The diagnosis of DED is challenging due to the poor correlation between symptoms and signs, and a correct diagnostic approach is fundamental to choosing a specific and effective treatment, with the main goal being to restore the tear film and ocular surface homoeostasis [7,9].

Different approaches are available for the treatment of ocular surface disorders, but tear substitutes represent the mainstay of the therapeutic approach to DED, with the aim being to restore the unstable tear film and to equilibrate its different layers in terms of quality and quantity [7,10,11,12,13]. For these purposes, the new-generation products are designed with combined formulas and are targeted to address and break specific points within the DED vicious cycle [14,15,16,17].

Multiple formulation eye drops, which are able to act at the same time on different etiological elements of DED, could enhance bioavailability, patients’ compliance and, consequently, treatment efficacy.

Recently, a new multiple-action ophthalmic solution composed of a mixture of elements acting specifically on different tear film layers with cytoprotective and anti-osmotic effects was introduced. Trimix ^®^ (Off Health Italia, Florence, Italy) is a protective, moisturizing and lubricating eye drop based on cross-linked hyaluronic acid (HA), trehalose and positively charged liposomes with cationic lipids, formulated for the restoration of the physiological properties of the ocular surface.

The combination of cross-linked hyaluronic acid and trehalose confers to the product a high viscoelasticity, a high degree of hydration and permanence on the ocular surface to relieve symptoms associated with dry eye. Several studies reported the clinical efficacy and functionality of each molecule present in this new formulation in the treatment of the ocular surface disorders [14,15,16,17,18,19,20,21,22,23,24]. The presence of different molecules, with their specific properties, allows for multiple-action activity over the ocular surface.

A study published by Vigo et al. reports the efficacy of this new formulation in treating DED patients, with an improvement of signs and symptoms after two months [25].

In this pilot study, we aimed to assess the efficacy, tolerability and safety of the new formulation versus a solution containing 3% trehalose alone (Thealoz^®^, Thea Pharmaceuticals, France) in subjects with moderate to severe dry eye to investigate the role of the additional molecules in the multiple-action new formulation.

## 2. Materials and Methods

In this single-center, prospective, single masked, observational cohort study, forty-one adult subjects diagnosed with moderate or severe dry eye (DEWS II classification) were enrolled [8].

All patients were at the first diagnosis, and no previous therapy was reported. The study was approved by the Ethical Committee for Clinical Investigation of Messina and was conducted at the Ocular Surface Diseases Center of Ophthalmology Clinic of University Hospital of Messina according to the Declaration of Helsinki. The inclusion criteria were age >18 years, Tear Break Up Time (TBUT) < 5 s, Schirmer test < 5 mm, presence of damage to the corneal epithelium, as evidenced by fluorescein stain, and Best Corrected Visual Acuity (BCVA) ≥ 0.3 logMAR in both eyes. The exclusion criteria were glaucoma, previous herpes virus, bacterial and mycotic corneal infections, corneal ulcers, three months history of conjunctival and lid infections, ocular surgery less than six months before the initiation of the study, history of ocular allergy and/or allergic rhinitis, systemic diseases, ocular therapies and systemic therapies potentially impacting ocular surface homeostasis.

Informed written consent was obtained from all participants, who were divided into two age- and sex-matched groups. Group 1 included 20 subjects (F 19 and M 1) aged 48 to 75 years (mean 66.25 ± 7.81 SD) who received the CXHAL solution, and group 2 comprised 21 individuals (F18 and M 3) aged 46 to 77 years (mean 67.29 ± 8.35 SD) treated with TRS eye drops.

The eye drops were administrated four times daily for 2 months, and all participants were evaluated at baseline, day 0 (V0) and day 60 ± 3 (V1) by two masked examiners.

The patients’ assessment included Best Corrected Visual Acuity (BCVA) in logMAR and Symptom Assessment in Dry Eye (SANDE) score for frequency and severity. Tear osmolarity was evaluated with the TearLab Osmolarity System (TearLab, San Diego, CA, USA). Keratograph 5M (Oculus, Wetzlar, Germany) was used to perform meniscometry, tear meniscus height (TMH), conjunctival hyperemia and, after the instillation of fluorescein, tear break-up time (TBUT), corneal and conjunctival fluorescein staining classified with the Oxford score and Meibography. For the assessment of these parameters, the automated grading system of Keratograph was used. Furthermore, slit lamp examination (Topcon SL 7D, Tokyo, Japan) was performed to evaluate eyelid redness, swelling and the presence of debris on the tear film using a score system from 0 (absent) to 3 (severe) to assess each parameter. 

### Statistical Analysis

The statistical analysis was performed using SPSS 26.0 for Windows statistical software (SPSS Inc., Chicago, IL, USA).

All numerical variables were expressed as the median (p50), 25% and 75% percentile (p25 and p75, respectively), and they were used to express the numerical variables for statistical purposes used to describe the characteristic of the sample. First, the Kolmogorov–Smirnov test was applied to verify if the examined variables presented a Gaussian distribution.

Successively, to test the treatment effect between the two times of the study, the Wilcoxon sign rank test was performed. Finally, only for parameters that improved significantly in both groups, the Mann–Whitney U-test was performed to test if there were significant differences between the two treatments. A *p*-value smaller than 0.05 was considered statistically significant.

## 3. Results

All enrolled patients completed the study. The BCVA and questionnaire results are reported in Table 1 and Table 2.

In Group 1, treated with the CXHAL solution, all questionnaire scores improved significantly, whereas, in Group 2, only the SANDE severity score showed significant improvements, the SANDE frequency being unvaried.

The results of the ocular surface parameters examined at baseline (V0) and after the treatment (V1) are represented in Table 1 and Table 2, respectively, for groups 1 and 2.

The unvaried parameters in both groups were BCVA, eyelid edema, TMH and Meibography. The parameters that improved only in the CXHAL group were SANDE frequency, eyelid erythema and osmolarity. The parameter that improved only in the TRS group was chemosis. The parameters that improved significantly in both groups were SANDE severity, hyperemia, TBUT and staining.

The further statistical analysis was performed only for those parameters that changed significantly in both groups to test if there were differences between the quantity of change in the two treatments. The amount of improvement registered in group 1 was compared with that registered in group 2. The quantity of improvement was equal in both groups for SANDE severity, hyperemia and corneal and conjunctival staining (*p* > 0.05). A significantly higher gain in the TBUT value was observed in group 1 vs. group 2 (*p* < 0.041).

Both eye drops were well tolerated, and no adverse events were reported during the study.

## 4. Discussion

DED is a very common disorder of the ocular surface characterized by tear film instability, hyperosmolarity, ocular surface inflammation and neurosensory abnormalities, with a consequent loss of homeostasis of the tear film that plays a pivotal role in its etiopathogenesis [1,8,12,26]. In relation to the severity of the disease, the patients complain of visual instability, foreign-body sensation, dryness and irritation. Due to the DED global impact, several authors focused their studies on the pathogenesis of the disease, with the aim to develop new therapeutic approaches. The target for the therapy is to restore the homeostasis of the ocular surface and improve the tear film layers and epithelial cells integrity and to block the DE vicious circle at its different points. The multifactorial aspect of DED induced the research for tear substitutes capable of acting on different pathogenetic points [7,10,11].

Trimix^®^ eyedrop is a new-generation multiple-action formulation containing 0.15% cross-linked HA, 3% trehalose and liposomes with cationic stearylamine. This ophthalmic solution is aimed to act directly on different points of the DED vicious circle.

HA is still the most used molecule among the tear substitutes, and its role in the treatment of DED is universally recognized [13]. It increases tear film thickness, stabilizes the muco-aqueous layer, lubricates, enhances visco-elasticity and promotes epithelial cells motility, proliferation and adhesion binding to the specific receptors on the epithelial cells [13,16,17]. Cross-linked HA (CX-HA), as a tear supplement, proved to provide a better ocular comfort than linear HA and therefore promoted a growing interest and diffusion [14,15].

As compared to the linear HA, the CX-HA showed a higher permanence on the ocular surface, a better distribution with blinking, higher support for the reparative processes and improved bioavailability and resistance to degradation [14,15].

Trehalose is a disaccharide with proven stabilizing and protective properties on cell membranes that prevents protein denaturation and lipid degradation and reduces inflammatory phenomena [18]. Trehalose has antioxidant and anti-inflammatory properties and osmoprotective and cytoprotective activity and maintains phospholipid integrity, so it can directly improve the condition of the affected eyes by hindering the dry eye “vicious circle” at multiple points. Trehalose promotes the restoration of tear film homeostasis and minimizes the chances for a return to the adverse condition [19,20,21].

Liposomes are vesicular systems composed of an aqueous core and surrounded by a lipid bilayer [22]. Their characteristics such as size, charge and lipid composition can be easily manipulated, which makes them versatile as nanocarriers for ocular drug delivery. Moreover, they exhibit properties such as low toxicity, biodegradability and the potential to encapsulate a variety of drugs with different features. Liposomes are known for their soothing and emollient properties, as well as for their ability to act as a vector, increasing the bioavailability of the carried substances and stabilizing the lipid component of the tear film [23]. On the ocular surface, liposomes anchor the lipid phase to the aqueous one, resulting in reduced evaporation and increased lubrication.

Sterylamine is a cationic lipid that provides a positive charge to the liposomes [24]. Due to the electrostatic interaction with negatively charged mucins, it ensures a prolonged residence of the formulation on the ocular surface and results in improved muco-adhesion and bioavailability with increased tear film stabilization. Additionally, cationic lipids exhibit anti-inflammatory properties that could represent a new therapeutic perspective in the treatment of ocular surface inflammation in the future [24].

In this pilot study, we aimed to compare the clinical effects of a new combined multiple-action eye drop (Trimix^®^) containing 3% trehalose with a solution of 3% trehalose alone to investigate the role of the additional elements, such as cross-linked HA and cationic liposomes with sterylamine, on the ocular surface in moderate–severe dry eye. Both tear film substitutes were safe and well tolerated and significantly improved SANDE severity, hyperemia, TBUT and staining. Trimix^®^ performed better, with an additional significant improvement in SANDE frequency, tear osmolarity and eyelid erythema. Moreover, the additional statistical analysis showed a significantly higher amount of TBUT increment in patients treated with Trimix^®^ as compared to the group treated with 3% trehalose alone.

This significantly better performance of Trimix^®^ could be attributed to its composition and its ability to improve tear film characteristics [14,15,21,22,23,24]. In this innovative eyedrop, the trehalose acts in close collaboration with cross-linked HA and liposomes. Trehalose is known to reduce oxidative stress and inflammation and has cytoprotective properties that resulted, in this study, in a statistically significant reduction in staining, hyperemia and TBUT in both groups [18,19,20,21].

A finding that must be particularly emphasized is the significant reduction in the osmolarity in the Trimix^®^ group. As hyperosmolarity is recognized as a principal element of the vicious circle of DED pathology, the significant improvement in osmolarity may explain the outcome, such as the amelioration of overall SANDE and the significantly higher improvement in BUT compared to trehalose patients [26].

Trehalose remains a fundamental element of therapies of the ocular surface disorders. In light of this study, the possibility to use the drops based on multiple molecules added to trehalose offers a more complete approach to treating patients with moderate and severe alterations of all tear film components and epithelial cells damage.

It can be concluded that the multifactorial nature of DED requires complex treatment, and the combined multiple-action formulation that targets different points of the DED vicious circle represents the best therapeutic approach and optimizes patients’ compliance. The Trimix^®^ eyedrop currently represents a new-generation all-in-one tear film substitute with a wide range of actions on the ocular surface, offering a new therapeutic approach in the treatment of moderate and severe dye eye disease. The limitations of this study are the relatively small number of participants and the fact that only two examinations were performed: at baseline and after 2 months. They are both related to the COVID pandemic period, which was accompanied by significant difficulties with patients’ recruitment and their accesses to the hospital.

Therefore, further clinical studies performed on a higher number of participants with more frequent assessments, investigating the role of single components, would be of great importance.

## Figures and Tables

**Table 1:** The BCVA and SANDE questionnaire results in both groups of treatments. Numerical variables are expressed as the median (p50), 25% (p25) and 75% (p75) percentiles. Statistically significant *p* values are highlighted in bold.

**CXHAL**	**Baseline V0**			**V1**			***p*-Value**
	**p25**	**p50**	**p75**	**p25**	**p50**	**p75**	
BCVA	−0.19	0	0.08	−0.15	0	0.02	0.969
SANDE frequency	27.5	51	80	25	36.5	51	**0.020**
SANDE severity	34	65	82	14	23	63	**0.001**
**TRS**	**Baseline V0**			**V1**			***p*-Value**
	**p25**	**p50**	**p75**	**p25**	**p50**	**p75**	
BCVA	−0.1	0	0	−0.1	0	0	0.157
SANDE frequency	41	60	82	30	51	71	0.222
SANDE severity	40	58	70	25	30	70	**0.012**

Bold indicate statistically significant *p*-values.

**Table 2:** Descriptive Statistics for both treatments and *p*-value for Wilcoxon sign rank test. Numerical variables are expressed as the median (p50), 25% (p25) and 75% (p75) percentiles. Statistically significant *p* values are highlighted in bold.

**CXHA**	**Baseline V0**			**V1**			***p*-Value**
	**p25**	**p50**	**p75**	**p25**	**p50**	**p75**	
Eyelid Erythema	1	2	3	1	2	2	**0.035**
Eyelid Edema	1	1.5	2	1	2	2	0.297
Hyperemia	0	1	1.5	0	1	1	**0.022**
Chemosis	0	0	0.5	0	0	1	0.803
TBUT	1	2	3	2	3	5	**<0.001**
Staining	1	1.5	2	1	1	1	**0.004**
Osmolarity	299	307.5	318	292	301.5	308	**0.001**
TMH	0.22	0.28	0.35	0.24	0.27	0.33	0.293
Meibography	2	2	2	1	2	2	0.206
**TRS**	**Baseline V0**			**V1**			***p*-Value**
	**p25**	**p50**	**p75**	**p25**	**p50**	**p75**	
Eyelid Erythema	2	2	2	1	2	2	0.091
Eyelid Edema	1	1	2	1	2	2	0.229
Hyperemia	1	1	1	1	1	1	**0.046**
Chemosis	0	0	0	0	0	0	**0.005**
TBUT	2	2	3	2	3	4	**<0.001**
Staining	1	2	3	1	1	2	**0.001**
Osmolarity	298	310	319	298	305	315	0.165
TMH	0.18	0.28	0.39	0.19	0.25	0.34	0.920
Meibography	1	2	2	2	2	2	0.989

Bold indicate statistically significant *p*-values.

## Data Availability

The data presented in this study are available on request from the corresponding author.
